# Control of IGFBP-2 Expression by Steroids and Peptide Hormones in Vertebrates

**DOI:** 10.3389/fendo.2014.00043

**Published:** 2014-04-07

**Authors:** Andreas Hoeflich, Elisa Wirthgen, Robert David, Carl Friedrich Classen, Marion Spitschak, Julia Brenmoehl

**Affiliations:** ^1^Institute of Genome Biology, Leibniz Institute for Farm Animal Biology (FBN), Dummerstorf, Germany; ^2^Ligandis GbR, Gülzow, Germany; ^3^Reference and Translation Center for Cardiac Stem Cell Therapy, Rostock, Germany; ^4^Pediatric Oncology, University Children’s Hospital, Rostock, Germany

**Keywords:** IGFBP-2, steroids, estradiol, peptide hormones, vertebrates

## Abstract

IGFBP-2 (1) has been described as a brain tumor oncogene (2) and is widely expressed in cancers from different origins (3–8). IGFBP-2 alone cannot cause malignant transformation, yet progression of brain tumors to higher grade (9) and also has been provided as a protective element in earlier stages of multistage colon carcinogenesis (10). Therefore, it is crucial to understand the factors that determine expression patterns of IGFBP-2 under normal and malignant conditions. The present review provides a comprehensive update of known factors that have an impact on expression of IGFBP-2.

## Introduction

Shortly after the identification of IGFBP-2 (1), it was realized that its expression both in liver and ovary is induced by E2 (Table 1) in hypophysectomized rats (11). Notably, hypophysectomy increased IGFBP-2 mRNA levels in the liver but decreased IGFBP-2 gene expression in the ovary. In the following 10 years of IGFBP-2 research, particular interest was taken in the somatotropic control of IGFBP-2 (8). However, it also was realized soon, that the gonadotropic axis may play a particular role in the control of IGFBP-2 expression (12). Just recently, Foulstone and coworkers have identified a feedback loop between IGFBP-2 and the ERa (estrogen receptor-alpha), whereby both factors can mutually induce gene expression of each other (13). This particular finding further underlined the current need for a detailed meta-analysis that is dedicated to the question which factors presently are known to control expression of IGFBP-2?

**Table 1:** **Cell- and tissue-specific control of IGFBP-2 expression in vertebrates**.

Tissue/cell type	Species	Factor	Effect	Reference
Brain	ch	Fasting	−	(14)
Astroglial cells	ra	IGF-I/II, insulin	+	(15)
Cortex	rat	E2, lithium	−	(16, 17)
Hippocampus	ra, mo	E2	+	(18, 19)
ctd	ra	Stress	−	(20)
Hypothalamus	ra	E2	+	(21, 22)
Pituitary	ov, pi, ra	E2	+	(18, 23–25)
Ovaries	ra, mo	DES, SSAT	+	(11, 26)
Follicle	bo, ov, ho,	FSH, P4 GHRH, leptin	+	(27–29)
ctd	fi	Leptin, E2, FSH, insulin	+	(30–32)
ctd	hu	A, LH, activin, IFγ	+	(33, 34)
ctd	bo, fi	PGF2a, IGF-I, E2	−	(27, 35, 36)
Uterus	pi, ra, mo	P4, relaxin, E2, SSAT	+	(26, 37–39)
Endometrium	hu, ap	E2, P4	+	(40–42)
Vagina	mo	E2	+	(43)
Breast	hu, mo, ra	IGF-I/PI3K, E2	+	(43–45)
Cancer	hu	IGF-I/PI3K, E2, P4	+	(46–48)
Cancer	hu, ra	E2	−	(49–51)
Prostate	ra, hu	Antiandrogen	+	(52, 53)
Stromal	hu	T and DHT	+	(54)
Cancer	hu	DHEA, T, DHT, E2	+	(55, 56)
Cancer	hu	A, vitamin D	−	(57–60)
Leydig cells	ra	hCG	−	(61)
Liver	ra, mo	DES, SSAT, leptin, fasting	+	(11, 26, 62–67)
ctd	ov, ra	T, insulin	−	(68–70)
Bone	ra	Mechanical loading	+	(71)
Kidney	hu, mo	IGF-I, SSAT	+	(26, 72)
Lung	hu	NF-kB, NeuroD, RA, O_2_	+	(73–77)
Cancer	hu	IGF-I	+	(78)
Spleen	ra	GH	+	(79)
Colon	hu	Vitamin D	−	(80)
Retina	ra	O_2_	+	(81)
Gizzard	ch	Insulin	−	(67)
Fin	fi	Androgen	+	(82)
Myoblasts	mo	IGF-I	+	(83)
Fibroblasts	hu	E2	+	(84)

*hu, human; ap, ape; pi, pig; ov, ovine; bo, bovine; ra, rat; mo, mouse; co, cony; ch, chicken; fi, fish; E2, estradiol; P4, progesterone; DES, diethylstilbestrol; SSAT, spermidine/spermine *N*1-acetyltransferase; FSH, follicle stimulating hormone; GHRH, GH releasing hormone; PGF, prostaglandin F; DH/T, dihydro/testosterone; DHEA, dehydroepiandrosterone; RA, retinoic acid; LH, luteinizing hormone; IFγ, interferon gamma; hCG, human chorionic gonadotropin; ctd, continued*.

## IGFBP-2 Promoter Region

First of all, tissue- and stage-specific expression patterns of IGFBP-2 are defined by endogenous stimulatory or repressor promoter elements within the IGFBP-2 gene. The promoter region of the gene encoding IGFBP-2 contains putative Sp1 binding sites (1, 85–87), potential response elements for E2 and P4 receptors, and overlapping sequences for albumin D-box binding (88). Kwak et al. have studied the structural basis for the stage-specific expression of IGFBP-2 in the porcine endometrium during pregnancy and identified two novel *cis*-elements in the promoter region of the IGFBP-2 gene (89). In this study, an endometrial 34-kDa nuclear binding protein was characterized with potential repressor functions for IGFBP-2 gene expression. These might confer site- and stage-specific expression of IGFBP-2 during pregnancy. Furthermore, a distal enhancer-like region, identified earlier in hepatic HepG2 cells (90) is active in the porcine endometrium (89). Four putative binding sites for NF-kB have been identified in lung alveolar epithelial cells isolated from rats that were exposed to oxygen (73). This finding by Cazals and coworkers may provide the functional basis for the effects of hyperoxia or hypoxia on IGFBP-2 expression described so far (74–76, 81). In small cell lung cancer cell lines, a proximal E-box was identified that binds NeuroD and thereby induces IGFBP-2 expression (77). In 11 out of 12 primary small cell lung cancer tissues assessed, the IGFBP-2 promoter was present in an unmethylated form, which confers higher gene expression in comparison with other histological subtypes (77). Since NeuroD is expressed also in other neuroectodermal cells, NeuroD-dependent induction of IGFBP-2 expression was discussed also for retinoblastomas, medulloblastomas, or neuroblastomas (77). However, also prostate carcinoma cells are known to express NeuroD (91) and, as discussed further down, also IGFBP-2. In human breast cancer cells (MCF-7), Mireuta and coworkers could demonstrate that the proximal promoter region of IGFBP-2 is activated by an IGF-I/PI3K/AKT/mTOR-dependent manner via an increase of nuclear Sp1 (44). The authors discussed the potential use of Sp1 inhibitors particularly in cancers that highly express IGFBP-2. Two potential androgen binding sites have been identified via bioinformatic analysis of the region upstream from the IGFBP-2 transcription initiation site that may explain the effects of androgen treatment on IGFBP-2 gene expression as demonstrated in prostate cancer cells (57, 58).

## Control of IGFBP-2 Expression in the Brain

More than 20 years ago, Pons and Torres-Aleman found that E2 significantly increases protein levels of IGFBP-2 in cultures of hypothalamic neurons isolated from rats (21). *In vivo*, application of E2 to ovariectomized rats also increased IGFBP-2 immunoreactivity in tanycytes and other ependymal cells of the hypothalamus (22). On the other hand, P4 led to reduced IGFBP-2 protein levels in the apical membrane of tanycytes in the same model. In both reports, functional interaction of steroids and the IGF-system was discussed. Also in the anterior pituitary glands from sheep (23), pigs (24), or rats (25) E2 increased IGFBP-2 levels. A positive effect of E2 on IGFBP-2 mRNA expression was present in MtT/S and GH3 cell lines established from rat pituitary adenomas (18). Notably, E2 seems to be permissive for steady state IGFBP-2 levels in the anterior pituitary since reduction of estrogens by use of anastrozole in boars decreased expression of IGFBP-2 (92). In contrast, DHEA increased IGF-I expression in the hypothalamus of rats but did not affect expression of IGFBP-2 (93). Estradiol further increased expression of IGFBP-2 mRNA in the hippocampus of ovariectomized rats (18) or in the hippocampus from normal mice 1 h after injection (19). Interestingly, also mice subjected to neonatal isolation had reduced hippocampal IGFBP-2 mRNA expression in adulthood when exposed to restraint stress (20). During hormone replacement therapy, E2 downregulated IGFBP-2 mRNA in the frontal cortex of ovariectomized rats (16). IGFBP-2 mRNA expression was robustly down regulated also by lithium in primary cell cultures from rat cortices (17).

The important role of IGFBP-2 in malignant brain tumors, particularly glioblastoma, has already been mentioned. Reactivation of IGFBP-2 expression in glioblastoma multiforme was discussed in a context of defect astrocyte differentiation and PI3K/AKT activation (94–96). Conversely, a stable knockdown of IGFBP-2 resulted in decreased invasiveness, decreased saturation density of the cells *in vitro*, and decreased tumorigenicity in nude mice (97).

A strong inverse relationship between elevated IGFBP-2 levels and low p16^INK4a^ indicates a negative regulatory function of p16^INK4a^ for IGFBP-2 (98) similar to the negative correlation of IGFBP-2 mRNA and PTEN expression levels found both in glioblastomas and prostate cancers, implicating IGFBP-2 as a biomarker for PTEN status (95). This may be in concordance with an observation of E2-induced apoptosis of glioblastoma cells (99). The inverse relationship between IGFBP-2 and PTEN expression is not restricted to malignant cells and has been described also during osteoclast differentiation (100).

## Control of IGFBP-2 Expression in Female Reproductive Tissues

Hormone replacement therapy over 15 months to postmenopausal women revealed a negative effect of E2 on serum IGFBP-2 concentrations (101). Also in human adolescents with constitutional tall stature, E2 reduced serum IGFBP-2 concentrations 3 and 6 months after starting the therapy (102). In contrast, in aged women and men supplementation of sex steroids over a period of 6 months revealed no effect of a hormone replacement therapy or testosterone on IGFBP-2 serum levels (103). In human granulosa cells, LH, activin, and interferon gamma increased expression of IGFBP-2 (33) and insulin and androstendione increased secretion of IGFBP-2 into cell conditioned media (34). After the preovulatory surge of LH, when steroid levels are low, an inverse relationship between follicular IGFBP-2 concentrations and steroid (E2, androstendione and P4) concentrations has been described in sows already in 1992 (104). FSH increased whereas prostaglandin F2a decreased IGFBP-2 concentrations in bovine ovarian follicular fluid and low levels of IGFBP-2 in E2-active follicles suggested a role of IGFBP-2 for aromatase activity (27). By contrast, P4 treatment in cattle increased protein levels of IGFBP-2 in follicular fluids (28). In equine granulosa cells, both E2 and FSH induced expression of IGFBP-2 *in vitro* (30). Furthermore, in follicles, also the GH/IGF axis is involved in the control of IGFBP-2 expression. Application of growth hormone releasing hormone in cattle increased IGFBP-2 in the circulation and in the fluid from subordinated but not from dominant follicles (29). As described earlier for E2, also human recombinant IGF-I decreased IGFBP-2 mRNA expression and protein levels in granulosa cells and the oocytes from cultures bovine antral follicles in a stage- and dose-specific manner (35). In addition, granulosa cells from bovine follicles but not the oocyte have been shown to produce an IGFBP-2 protease (105). Thus, also IGFBP-2 protein stability is under active control in bovine follicles. Finally, leptin infusion for 3 days in cycling ewes increased follicular IGFBP-2 mRNA expression although this effect may be related to other hormones since at the same time insulin and FSH serum concentrations were increased while those of E2 were reduced (31).

IGFBP-2 secretion is stimulated by E2 and P4 in human endometrial stromal cells and in endometrial explants from baboons (40, 41). Short-term E2/P4 treatment of ovariectomized monkeys over 2 weeks increased IGFBP-2 mRNA in the myometrium in response to E2 and to a higher degree after treatment both with E2 and P4 (42). High amounts of IGFBP-2 mRNA are found in the porcine uterus (106), which might be related to the specific IGFBP-2 promoter configuration as summarized above (88). Progesterone increased gene expression of IGFBP-2 in pig uteri while E2 slightly reduced mRNA levels of IGFBP-2 (37). Notably, the trophic effects of relaxin administration on uterine weight in pigs were accompanied by robust increase of IGFBP-2 as found as a band doublet in uterine flushes (38). High IGFBP-2 levels in earlier but not in later phases of the estrus in pigs are potentially due to kallikrein/matrix metalloproteases (107). In fact, kallikrein, matrix metalloprotease 3, or plasminogen activator were sufficient to rapidly degrade IGFBP-2 in uterine flushes or breast milk from pigs (107) or humans (108), respectively. Six hours after E2 injection in mice, IGFBP-2 mRNA expression was increased in the mammary gland but even more in the vagina (43). Similarly, E2 treatment significantly increased IGFBP-2 mRNA levels also in uteri of ovariectomized rats (39). A robust increase of IGFBP-2 gene expression was further found in uteri from mice characterized by transgenic spermidine/spermine *N*1-acetyltransferase (SSAT) expression (26). Steroid control of IGFBP-2 is also observed in non-mammalian species as gonadotropin, E2, and P4 were able to increase expression of IGFBP-2 mRNA in de-yolked follicles from the rainbow trout (32) whereas E2 decreased IGFBP-2 mRNA expression in the orange-spotted grouper (36).

## Control of IGFBP-2 Expression in Breast Cancer Cells

In human breast cancer cells (MCF-7), IGF-I potently induced expression of IGFBP-2 (46) and E2 enhanced the effect of IGF-I with its basal activity being on a lower level if compared to IGF-I (47). Martin and Baxter further demonstrated that both the effects of IGF-I and E2 were mediated by the PI3/AKT pathway since inhibitors of IGF1R, PI3K, and mTOR blocked the basal effects of IGF-I and E2 (47). Also in mammary glands from rats, E2 increased mRNA expression of IGFBP-2 mRNA (45), while in pseudo-pregnant pigs E2 injection did not affect mammary IGFBP-2 mRNA expression (109). In invasive (MCF-7/6) or in breast cancer cell lines adapted to low serum concentrations (MCF-7/S0.5) (49, 50) and in Fischer rat mammary adenocarcinoma cells (51), E2s suppressed intracellular and/or secreted levels of IGFBP-2. Nevertheless, secretion of IGFBP-2 was lower in ER-negative breast cancer cells compared to ER-positive cells indicating a positive or at least a permissive effect of ER on IGFBP-2 expression in mammary cells (110). Therefore, similar to cells from the brain a synergistic effect of E2 and P4 on IGFBP-2 secretion was found in breast cancer explants (48). Since this was true only for hormone-sensitive but not for hormone-insensitive samples, it also supports at least a permissive role of ER for the functional relationships between E2- and P4-signaling on the one hand and IGFBP-2 expression on the other. Furthermore, IGFBP-2 is highly expressed by antiestrogen-resistant breast cancer cell lines (111) and in antiestrogen-resistant RU58R-1 cells, IGFBP-2 expression was suppressed by E2 but massively stimulated by pure antiestrogen (50). Therefore, an indirect effect of E2 and the interaction of estrogens and IGFs has been suggested (49). In fact, a dedicated review discusses the crosstalk between IGFs and E2 (112) with E2 being introduced as an enhancer of IGF-signaling pathways in breast cancer. This kind of view was further elaborated and thus supported by the work of Chan et al., who described significant activation of IGF1R, IRS-1 and -2, AKT, and PI3K in response to E2 in the rat mammary gland (45).

## Control of IGFBP-2 in Prostate Cells

In primary human prostate stromal cells and in human prostate cancer cells (LNCaP), androgens and in part also E2 significantly induced IGFBP-2 mRNA expression (54–56). Flutamide, an androgen receptor antagonist, blocked androgen-dependent induction of IGFBP-2 expression (54). Also antisense oligonucleotides or antiandrogen treatment reduced gene expression of IGFBP-2 in LNCaP cells (113). It was speculated, that the effects of DHT and T are mediated by the AR. Looking at the level of protein, it was demonstrated that androgen treatment rapidly decreased IGFBP-2 levels in LNCaP cells (59). Notably, negative androgen regulation of IGFBP-2 secretion involved extracellular proteolytic cleavage (59). A reduction of IGFBP-2 proteolysis in androgen-insensitive prostate carcinoma cells increased the metastatic potential in that study. Androgen-dependent regulation of IGFBP-2 mRNA expression might be established by means of the IGFBP-2 promoter specifically, as stated earlier, but also by an effect on global protein translation: unexpectedly, androgen treatment of prostate carcinoma cells for an extended period of time (48 h) downregulated the polysomal fraction of mRNA and thus global protein synthesis (57). Further supporting negative effects of androgens, castration induced gene expression of IGFBP-2 in the rat ventral prostate (114). Also antiandrogenic treatment increased tissue levels of IGFBP-2 in human patients (52) or in rats (53). Therefore, reports are available that demonstrate or suggest positive or negative (58) effects of androgens both on mRNA and protein levels of IGFBP-2 depending on the physiological condition in prostate cells.

## Control of IGFBP-2 by GH and IGFs

Zapf et al. performed the initial study on hormonal control of IGFBP-2 by GH and IGF-I (115). In this study, GH suppressed expression of IGFBP-2 while IGF-I increased this particular IGFBP as demonstrated by Western ligand blotting. Notably, GH suppressed the effect of IGF-I on IGFBP-2 concentration. After development of a specific radioimmunoassay by Blum and coworkers (116), higher levels of IGFBP-2 have been quantified during GH deficiency and IGF-I administration whereas reduced IGFBP-2 levels were present in acromegalic patients. An inverse relationship between IGFBP-3 and IGFBP-2 was diagnosed (116), indicating that GH is a suppressor of IGFBP-2 expression. This finding was repeatedly confirmed by others and may represent the basis for altered IGFBP-2 levels during fasting, in aging, or impaired liver function as suggested, e.g., by Bannink and coworkers (117). Exogenous GH downregulates IGFBP-2 in humans (117–119), whereas impaired GH receptor signaling in mice (120) elevated IGFBP-2 serum levels. Although not confirmed as a rule (121, 122), the strength of the inverse relationship between GH and IGFBP-2 and its bidirectional nature suggested the use of IGFBP-2 levels as a biomarker to monitor GH-doping (123) or IGF/IGFBP-3 misuse in male and female athletes (124). Interestingly, testosterone blocked the negative effects of growth hormone on IGFBP-2 levels in men (103) and in GHR-deficient mice testosterone increased serum levels of IGFBP-2 (125). Conditional effects of GH have been described with more suppressive effects on IGFBP-2 levels in lean versus obese sheep (126). To date, exclusively in the spleen from juvenile rats exogenous GH stimulated gene expression of IGFBP-2 in that particular tissue (79).

IGF-I increased IGFBP-2 in human subjects (121, 127), in human embryonic kidney fibroblasts (72), in human lung adenocarcinoma cells (78), in transgenic rabbits (128), in rat astroglial cells (15), or in mouse C2C12 myoblasts (83). Administration of pegylated IGF-I in mice increased serum levels of IGFBP-2 up to 30 μg/ml, which corresponds to an increase of 1–2 magnitudes (129). Positive correlations of serum levels between IGF-II and IGFBP-2 have been frequently observed, e.g., in human subjects (116) or in IGF-II transgenic mouse models (130, 131). Thus, also IGF-II has been considered as a major regulator of IGFBP-2 expression. In the medulla oblongata of IGF-II transgenic mice, protein levels of IGFBP-2 were 10-fold upregulated if compared to non-transgenic mice (132). IGF-II also increased secretion of IGFBP-2 by primary rat astroglial cells (15). An interaction of IGF-I and estrogens has been discussed in women with anorexia nervosa (133). In that setting, E2 reduced IGF-dependent increase of IGFBP-2, which was discussed in a context with higher levels of free IGF-I in the presence of estrogens.

## Control of IGFBP-2 Expression by Diet and Insulin

IGFBP-2 has been provided as an antidiabetic and antiobesity protein by the pioneer work of Wheatcroft and coworkers (134). This finding was confirmed and leptin was shown also to stimulate hepatic expression of IGFBP-2 (62). Accordingly, an important role of IGFBP-2 for metabolic homeostasis has been discussed (135). On the other hand, in humans serum concentrations of IGFBP-2 can be increased by protein or carbohydrate intake (136–138). In addition, also single supplementations, e.g., lycopene in humans (139), or vitamin D analogs in cancer cell lines (60, 80) can induce or repress IGFBP-2 concentrations. Serum levels of IGFBP-2 were positively associated with insulin infusion in humans (140) and negatively correlated in dairy cows (141, 142). With exceptions (121), fasting increased serum levels of IGFBP-2 from humans to chicken and re-feeding normalized high fasting IGFBP-2 serum levels (130, 143–146). So far, in catfish, fasting did not affect IGFBP-2 expression (147). Altered serum levels may be due to hepatic expression of IGFBP-2 mRNA, which is increased in fasted or diabetic rats (63–66). Also in chicken, fasting increased IGFBP-2 expression in the liver and gizzard, and insulin administration decreased IGFBP-2 expression in both tissues (67). By contrast, in the brain, fasting efficiently reduced expression of IGFBP-2 mRNA as shown by Kita and coworkers in chicken (14). Insulin suppression of IGFBP-2 expression was also observed in hepatocytes isolated from rats (68, 69). However, dietary control of IGFBP-2 expression seems to occur also in non-hepatic cells, since insulin significantly increased mRNA expression of IGFBP-2 in primary rat astroglial cells (15). Nutritional regulation of IGFBP-2 also expands into the ovaries from sheep, where dietary factors (infusion of glucose and glucosamine or lupine supplementation) increased its expression in follicular granulosa cells. Thereby, higher IGFBP-2 levels correlated with the number of atretic follicles (148).

## Summary and Conclusion

Expression of IGFBP-2 is depending on tropic signals from the IGF/PI3K pathway, dietary factors, and oxygen. However, in a wide variety of tissues, steroids have been identified as effectors of IGFBP-2 expression. Steroids may impact on the level of IGFBP-2 mRNA and protein expression or stability. In addition to reproductive organs particularly within the brain steroids have major effects on the expression of IGFBP-2. Above all other steroids, E2 appears to have a particular function for the control of IGFBP-2 levels. Furthermore, in different tissues E2 seems to modulate IGF-signaling pathways. With respect to IGFBP-2 levels, interactions of GH/IGF-signals and steroid signals seem to exist on the level of cytosolic signal transduction but also on the level of RNA transcription within the cell nucleus. Those interactions might provide novel molecular targets in the prevention or therapy of malignant or metabolic diseases (2–7). Looking back at 25 years of IGFBP-2 research, the dominant role of E2 for regulation of IGFBP-2 activity is intriguing. To date, the understanding of functional interrelations between IGFBP-2 and steroids is likely just in its beginnings.

## Conflict of Interest Statement

Dr. Elisa Wirthgen is part of the Ligandis GbR team. This relation has no effect on the content of the manuscript. The other co-authors report no conflicts of interest.

## Abbreviations

AR, androgen receptor; AKT, protein kinase B; DHEA, dehydroepiandrosterone; E2, 17β-estradiol; ER, estrogen receptor; FSH, follicle stimulating hormone; GH, growth hormone; GHR, GH receptor; IGF, insulin-like growth factor; IGFBP, IGF binding protein; IGF1R, IGF-I receptor; LH, luteinizing hormone; mTOR, mammalian target of rapamycin; PTEN, phosphatase and tensin homolog; P4, progesterone; T, testosterone.
